# The properties of hot household hygroscopic materials and their potential use for non-medical facemask decontamination

**DOI:** 10.1371/journal.pone.0255148

**Published:** 2021-09-07

**Authors:** Marie-Line Andreola, Fréderic Becquart, Wahbi Jomaa, Paul O. Verhoeven, Gérard Baldacchino, Simon Hemour, D-Mask consortium

**Affiliations:** 1 Université de Bordeaux, MFP, CNRS UMR 5234, Bordeaux, France; 2 UB’L3 TBM core, Bordeaux, France; 3 Université de Lyon, CNRS, UMR 5223, Ingénierie des Matériaux Polymères, Université Jean Monnet, Saint-Etienne, France; 4 Université de Bordeaux, CNRS UMR 5295, Bordeaux, Talence, France; 5 CIRI, Centre International de Recherche en Infectiologie, Equipe GIMAP, Université de Lyon, Université Jean Monnet, INSERM U1111, CNRS, ENS, UCBL1, St‑Etienne, France; 6 Laboratory of Bacteriology-Virology-Hygiene, University Hospital of Saint-Etienne, Saint-Etienne, France; 7 Université Paris-Saclay, CEA, CNRS, Laboratoire Interactions, Dynamique et Lasers (LIDYL), Gif-sur-Yvette, France; 8 Université de Bordeaux, CNRS UMR 5218, Bordeaux INP, IMS, Talence, France; VIT University, INDIA

## Abstract

The widespread use of facemasks throughout the population is recommended by the WHO to reduce transmission of the SARS-CoV-2 virus. As some regions of the world are facing mask shortages, reuse may be necessary. However, used masks are considered as a potential hazard that may spread and transmit disease if they are not decontaminated correctly and systematically before reuse. As a result, the inappropriate decontamination practices that are commonly witnessed in the general public are challenging management of the epidemic at a large scale. To achieve public acceptance and implementation, decontamination procedures need to be low-cost and simple. We propose the use of hot hygroscopic materials to decontaminate non-medical facemasks in household settings. We report on the inactivation of a viral load on a facial mask exposed to hot hygroscopic materials for 15 minutes. As opposed to recent academic studies whereby decontamination is achieved by maintaining heat and humidity above a given value, a more flexible procedure is proposed here using a slow decaying pattern, which is both effective and easier to implement, suggesting straightforward public deployment and hence reliable implementation by the population.

## Introduction

Facemasks are being widely used amidst the global COVID-19 pandemic to reduce airborne virus transmission in the context of social interactions [1]. Indeed, high viral loads of severe acute respiratory syndrome coronavirus 2 (SARS-CoV-2) can be found in both asymptomatic [2] and positive patients [3]. Decontamination of used facemasks (textile and if possible FFP and surgical) is a common practice to enable their reuse and thus mitigate the shortage risk [4, 5], as well as the potential ecological impact of disposable units worn by billions of daily users [1, 6]. Slightly different from disinfection (which kills microorganisms on contaminated surfaces), decontamination reduces (typically, a 5 log reduction) the microbial contamination of materials or surfaces to an acceptably safe level to avoid contamination. However, the recommended household decontamination procedures are time- and energy-intensive [7], which can potentially lead to low public acceptance, and result in some cases in infrequent or slapdash decontamination practices. Since SARS-CoV-2 can remain on the mask’s surface for a long time (up to seven days on surgical masks [8] (chin AWH, Chu JTS, et al lancet microbe, 2020)), the mask itself can become a vector of contamination if it is not systematically decontaminated, thereby sustaining the spread of the epidemic. To contain this issue, an easy, low-cost, and quick decontamination procedure is therefore required to increase the daily usage of facemask decontamination.

Moist heat, a combination of heat and humidity, is a known treatment method for inactivating certain pathogens. For example, influenza viruses on stainless steel surfaces have been inactivated after having their temperature raised to 65°C for 15 minutes associated with a relative humidity (RH) of 25% [9], while equivalent levels of inactivation have been reached at 55°C and 75% RH. However, dry heat at 70°C may not be sufficiently effective to inactivate the virus, even over a longer exposure time of an hour [10]. Meanwhile, RH is the most widely reported parameter [11, 12]. In the combined heat and high humidity condition, the arrangement of the lipid bilayer of the virus as well as the interactions involved in the envelope proteins may be affected [13–15]. In droplets, the humidity rate and the evaporation kinetics of water can produce [12] an intermediate evaporation rate and a high concentration of salts, leading to virus inactivation. Unfortunately, all previous studies have been carried out in constant conditions over time, and these are not easily met in a domestic setting (constant temperature and humidity require sensors and a regulation system) [5].

With the aim of using the temperature-humidity synergy in a simple setup, we report here on SARS-CoV-2 virus inactivation on cloth masks in a box filled with hot hygroscopic materials available in household settings. After being heated separately by any heat source, the hot hygroscopic materials will release steam, and act as both a heat source and a humidifier for the closed box. The decontamination process follows a decaying temperature pattern (the thermal mass of the hygroscopic material surrounding the mask cools slowly) and does not involve regulation. As it cools down, the hygroscopic medium re-absorbs some of the moisture, which "re-activates" it and allows for numerous reuses.

## Materials and methods

All investigations in this work were performed on “Boldo’R" cloth masks from the Boldoduc Company, which is composed of three layers (layer 1 and 3: 100% cotton, layer 2: 100% polyester [reference: https://www.boldo-r.fr/], designed and manufactured as specified by AFNOR SPEC S76-001-2020. Heating tests were performed on the three types of short cut pastas detailed below.

### Inactivation assay conditions

**Fig 1:**
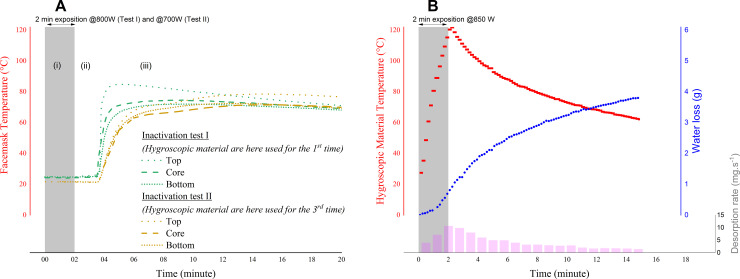
(A) Temperature evolution inside the mask under the same steaming conditions (sealed container) as those used for the biological trial shown in Fig 2. (B): Temperature, water loss, and water vapor desorption rate from the initial 500g of hygroscopic material during and after a household-compatible heat and humidity decontamination process (open container). The desorption rate is linked to the concentration of water at the surface of the medium, and thus its pattern is a direct image of the material’s temperature. The fast heating process does not cause the hygroscopic medium to release all the water it contains, because the mass loss is limited by the slow water mobility of the material.

Test I was performed at the GIMAP lab using Panzani pasta “coquillettes 8 min.” hygroscopic materials, “Boldo’R" cloth masks #1, #2, and #-3, and a MSO23 microwave oven (Hitachi, 800W RF power). For temperature measurement (Fig 1A), sensors (thermocouples, K type) were sewn onto the outer side of the cloth mask (“Top” and “Bottom” bottom), and sewn into the fold of the mask laid flat (“Core” data).

Test II was performed at the MFP lab using Barilla pasta “coquillettes 7 min.” materials, “Boldo’R" cloth mask #4, and a MWD 201/WH microwave oven (Whirpool, 700W RF power). For temperature measurement (Fig 1A), sensors (Thermo Buttons 22T) were introduced into the three fabric layers of the cloth mask. “Top” data corresponds to the interspace between the top and middle layer, “Bottom” data corresponds to the interspace between the middle and the bottom layer, and “Core” data corresponds to the fold, with a layer above and a layer below.

The data shown in Fig 1B were obtained with Lustucru pasta “coquillettes 3 min.” materials and a custom multimode microwave oven (850W RF power) allowing for real-time weight measurement. Since no rotating platform was available for this measurement, an area representative of typical household oven heating was selected. Temperature measurements were performed during and after the microwave heating using a fiber optic thermometer (Omega Company) introduced into the hygroscopic material stack.

### Inactivation studies

For each inactivation study, 15,000 VERO E6 cells were seeded in 24 well plates for 24 hours to reach 80% confluence. A 25 μl sample of viral solution (5.13 Log TCID50/ml for test I and 4.84 Log TCID50/ml for test II) was spotted on paper patches such as antibiotic discs (6mm-diameter) and dried at room temperature for 10 minutes. Since a new cloth mask (unwashed) contains few compounds that are toxic to the cells used for the biological assay, (see results & discussion part) patches (paper disks) containing the virus were introduced into the folds of a whole mask (to mimic the real conditions of decontamination of an entire mask). The mask and paper discs were put into a box and were then subjected to the heat and moisture decontamination procedure. After 20 minutes for test I and 15 minutes for test II, the patches were removed and incubated in a cell culture medium for another 15 minutes, and the eluates were used to infect the VERO cells. For test I, the virus was recovered from the paper discs by elution in 225 μl of fresh medium and 100 μl of this suspension was seeded onto the confluent Vero E6 cells. For test II, 500 μl of medium was used and 400 μl of this suspension was used to infect the Vero E6 cells. The cells were observed at day 2, 5, 6, 7 and 9 post-infection to monitor the appearance of the cytopathic effect. Cells were also fixed with PFA 4% for 20 min and stained in order to be imaged in BSL2. Images were acquired at 40x magnification. Each image corresponds to 9 merged fields which cover approximately 0.95 mm^2^.

#### Heating procedure

The following three-step procedure was tested (Fig 1A) (see S1 Movie of the overall decontamination process): Two 1L polypropylene containers, each filled with 250g of hygroscopic material, were first heated in a microwave oven for 2 minutes (i). The containers were taken out of the oven and the mask was placed in one of them, on top of the hot hygroscopic material, while the remaining material was poured onto the mask. As a result, the mask was sandwiched between two thick layers of hygroscopic materials (short cut pastas). This step was tested with a handling time below 1:30min (ii). The container was then hermetically sealed for 15–20 min to create a high-humidity atmosphere (iii).

## Results and discussion

During the first phase of the heating procedure, the porous hygroscopic medium was exposed to microwave energy. With this technique, heat is then generated inside the material throughout its volume, leading to faster heating rates compared to conventional heating, where heat is usually transferred from the surface to the interior. Heating efficiency depends on wave interactions with polar molecules or clusters [16] described by the loss factor, which is responsible for wave attenuation and the conversion of electrical field energy into heat. In the case of dry food products, they are strongly linked to residual moisture content and starch composition [17]. Wheat, corn and rice starches are typically used in microwave food product formulations [18], since the dielectric loss factors of these starches are commonly above 14, as compared to about 10 for water (data given at 20°C, [19]). These materials, such as short-cut pasta evaluated in this work, are considered as good household candidates for carrying and generating the heat and humidity required for the decontamination method.

**Fig 2:**
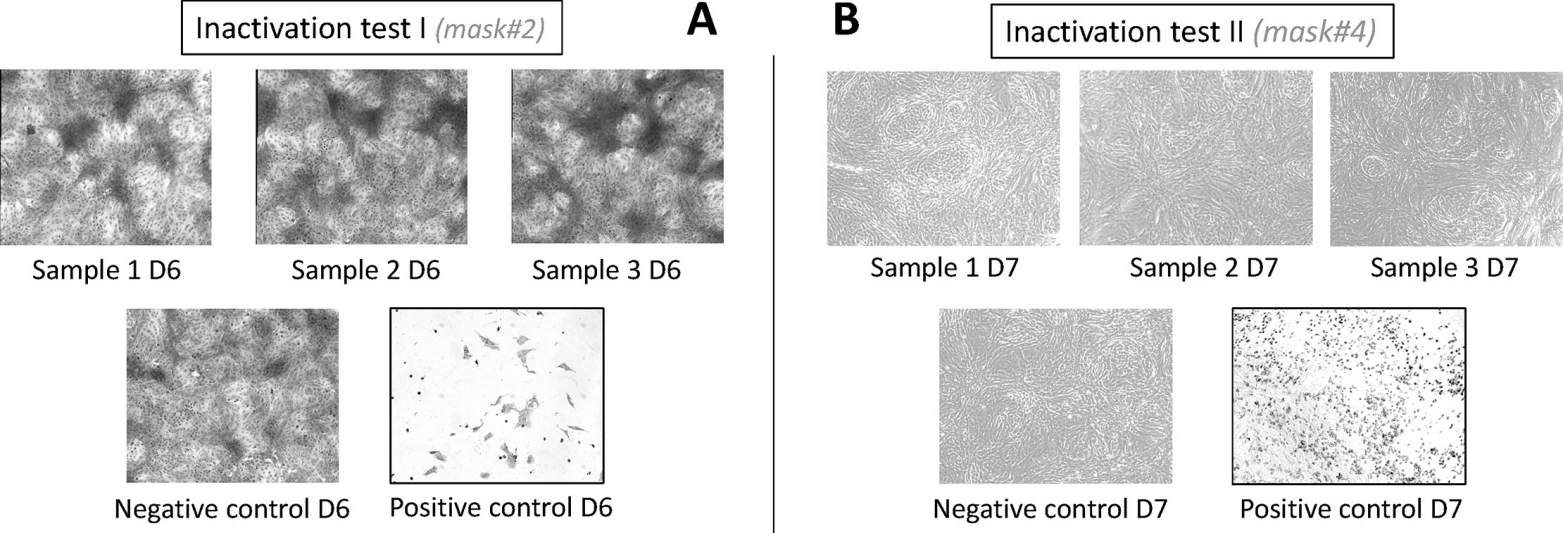
Effect of heat-inactivation on SARS-CoV-2 on the surface of a cloth mask *performed in two different laboratories (test I in UB’L3 TBM core and test II in CIRI GIMAP). Vero E6 cells inoculated with SARS-CoV-2 recovered from paper discs after heat-inactivation (Sample 1–3). Vero E6 growth with complete medium (negative control). Vero E6 cells inoculated with SARS-CoV-2 recovered from paper discs without heat-inactivation (positive control). The cytopathic effect was observed at Day #6 for test I and at day #7 for test II. The cells were growing in every case except for the positive control where a strong cytopathic effect was observed. (Results from four independent inactivation tests with a total of 18 samples available in the supplementary materials).

During the decontamination stage (iii), the mask carrying the virus was “sandwiched” inside the non-consolidated hygroscopic porous media. The relative low thickness of the mask and its low heat capacity compared to the heat capacity of the porous stack (675 J K-1 for 500 g of the chosen media, compared to 30 J K-1 for a cloth mask) ensured that a local thermal equilibrium was reached rapidly. As seen in Fig 1A, the evolution of the mask temperature was then governed by that of the porous hygroscopic media temperature. As the container was sealed during this period, heat and mass transfer with the environment were limited, leading to a slow cooling process and thus maintaining a high temperature. For the inactivation test II (shown in Figs 1A and 2), the temperature was maintained at a minimum of 70°C during a minimum time of 5 min. To assess the reliability of the procedure, we performed 20 other heating tests (not shown here) on five different household microwave ovens (700W to 900W), for which all temperatures were measured above the inactivation test II temperatures.

Due to its hygroscopic nature [20], a rise in temperature inside the pastas leads to water desorption and subsequent migration of vapor from the core of the material to its surface. The released vapor increases the relative humidity inside the sealed container in the vicinity of the mask. One can note that saturation (100% RH) is reached quickly as the additional vapor requirement is low compared to the desorption rate of the material (Fig 1B). Indeed, at 70°C, for example, saturated moist air has a specific volume of 1.4 m3/kg of dry air and contains 0.28 kg of vapor per kg of dry air (psychrometric chart). Therefore, in a 1L sealed box filled with 500 g pasta at 70°C, the volume occupied by air is almost equal to 0.65 L (for an intrinsic density of pasta equal to 1450 kg/m3) and is then saturated if it contains only 0.13 g of vapor.

The proposed method was thus not only able to raise the temperature of the mask to a high value (Fig 1A), but also to develop high humidity (Fig 1B) conditions for virus inactivation. In the hours following the experiment, the cooled hygroscopic porous medium gradually reabsorbed humidity from the environment (data not shown here). The phenomenon of adsorption is explained by the thermodynamic equilibrium of any hygroscopic material with the surrounding air, by means of sorption- and desorption- isotherm, and is well documented (see for instance water uptake in spaghetti pasta in [21]. As for the adsorption speed, it depends on the relative humidity of the ambient conditions. This can be seen as a self-reactivation mechanism which allows the material to be used for numerous further decontamination cycles. It was expected that over the decontamination cycles, heat-induced water loss from the hygroscopic medium would diminish, leading to a higher temperature. To evaluate this effect, the inactivation tests were performed with “new” materials (Test I) and materials used for the third time (Test II). Additionally, the two tests were carried out with different heating powers in different BSL3 laboratories, leading to two truly independent validations of the decontamination process.

Initial assays were performed by spotting uninfected medium or medium with viral suspension on cloth masks, but the medium was not easily absorbed due to the hydrophilic properties of the mask, and the recovery of the virus was difficult because of the large size of the textile piece needed to absorb 25 μl.

With brand new masks, the medium alone eluted from the textile of the brand new cloth mask had a cytotoxic effect on Vero E6 cells. After simple machine washing, this cytotoxic effect disappeared. Although the cloth masks are approved for wear, the textile is not compatible with cell culture applications. Some unrecognized toxic compounds seem to be released by the new textile into the culture medium, which made the experiments more complicated. Because of the mask shortage during the pandemic, it was difficult to have enough masks to cut into small pieces to optimize the absorption stage, leading to our choice of using paper discs. Therefore, viral supernatant was spotted on the paper discs, and the patches containing the virus were introduced into the folds of a whole mask, as described in the methods section. Three infections were carried out in parallel (samples 1, 2 and 3) for each mask. Paper discs spotted with 25 μl of culture media, either treated with hygroscopic materials or untreated, were used as a negative control. A positive control corresponding to the spotted but untreated virus was also performed.

While the cells with untreated virus (Fig 2, positive control) were lysed, those incubated with treated virus continued growing and did not show any cytopathic effect even 9 days post-infection, showing the efficacy of the treatment. No toxic product was released after treatment with hygroscopic materials (negative treated control). RNA from inactivation test II were also extracted and amplified by qRT-PCR. Three regions of the SARS-CoV-2 genome were targeted (N, and two regions of Orf 1ab). While the viral RNA was detected in the positive sample, as expected (Ct around 15,9), in the samples treated with the hygroscopic process it was undetected, in the same way as the negative control and the three amplified regions, corresponding to a loss of infectivity above 5 log.

## Conclusion

In all, our results indicate that the hot hygroscopic protocol described in this work provides reliable and stable heat and humidity conditions which are efficient for the inactivation of viral infectivity. The procedure decontamination is easy and effective and can be adapted to any type of mask in the most of the homes. Temperature homogeneity is achieved in this work with pasta, but other hygroscopic materials such as rice or dry seeds could be considered. This technique can be used to solve supply problems in the event of a pandemic or natural disaster. The possible recycling of the masks is in favor of the sustainability sought to limit waste and conserve resources. This protocol has significant potential to initiate new research into the “decaying” temperature and humidity decontamination techniques that are critical to the concept of simple and reliable operation in household settings.

## Supporting information

S1 Movie Video of the overall decontamination process including the step (i) to (iii) described in Fig 1 of the main manuscript. (MP4)

S1 Fig Vero E6 cells at day 6 post infection. VeroE6 cells inoculated with SARS-CoV-2 recovered from paper discs after heat-inactivation (mask #1). VeroE6 growth with complete medium (negative control). VeroE6 cells inoculated with SARS-CoV-2 recovered from paper discs without heat-inactivation (positive control). Images were acquired at 40x magnification. Each image corresponds to 9 merged fields which cover approximately 0.95 mm^2^.
(PDF)

S2 Fig Vero E6 cells at day 6 post infection. Vero E6 cells inoculated with SARS-CoV-2 recovered from paper discs after heat-inactivation (mask #2). Vero E6 growth with complete medium (negative control). VeroE6 cells inoculated with SARS-CoV-2 recovered from paper discs without heat-inactivation (positive control). Images were acquired at 40x magnification. Each image corresponds to 9 merged fields which cover approximately 0.95 mm^2^.
(PDF)

S3 Fig Vero E6 cells at day 6 post infection. Vero E6 cells inoculated with SARS-CoV-2 recovered from paper discs after heat-inactivation (mask #3). Vero E6 growth with complete medium (negative control). Vero E6 cells inoculated with SARS-CoV-2 recovered from paper discs without heat-inactivation (positive control). Images were acquired at 40x magnification. Each image corresponds to 9 merged fields which cover approximately 0.95 mm^2^.
(PDF)

S4 Fig Vero E6 cells at day 2 and 7 post infection. VeroE6 cells inoculated with SARS-CoV-2 recovered from paper discs after heat-inactivation (mask #4). VeroE6 growth with complete medium (VERO cells). Uninfected VeroE6 cells with paper discs after heat-inactivation (Negative control). VeroE6 cells inoculated with SARS-CoV-2 recovered from paper discs without heat-inactivation (positive control).
(PDF)

S5 Fig qRT-PCR amplification of viral RNA. Methods from the experiment shown in S4 Fig. After centrifugation, 200 μl of supernatant were extracted using the « High Pure Viral Nucleic Acid » kit from Roche following the supplier’s recommendations. qRT-PCR was performed following indications of the supplier (SARS-CoV-2 RT-qPCR Perkin Elmer). Briefly, 6 μl of reactional mix were mixed with 14 μl of a 1/10 dilution of each sample. Amplification of positive and negative controls supplied in the kit were also performed. Two viral genes, ORF1ab-gene and N-gene, were targeted. According to the supplier, Ct ≤32 for ORF1ab or ≤35 for N-gene are considered as positive. Ct ≥40 or undetermined are negative. S5 Fig presents a representative experiment of qRT-PCR. Supernatant of cells infected with positive control (untreated virus) were positive with Ct of 15.80 and 16.10 for ORF1ab-gene and N-gene respectively. All other samples, uninfected cells, cells infected with heat treated samples, negative control of the kit, were undetected. Positive controls of the kit is detected with Ct ≤32 as expected: 29.25 for of ORF1ab and 28.92 for N-gene.
(PDF)
