# Supplementary material for: The properties of hot household hygroscopic materials and their potential use for non-medical facemask decontamination
Source: PLoS One. 2021 Sep 7;16(9):e0255148. doi: 10.1371/journal.pone.0255148 (PMC8423240; doi:10.1371/journal.pone.0255148)
Supplement: S3 Fig — Vero E6 cells inoculated with SARS-CoV-2 recovered from paper discs after heat-inactivation (mask #3). Vero E6 growth with complete medium (negative control). Vero E6 cells inoculated with SARS-CoV-2 recovered from paper discs without heat-inactivation (positive control). Images were acquired at 40x magnification. Each image corresponds to 9 merged fields which cover approximately 0.95 mm2. (PDF) [file pone.0255148.s004.pdf]

Marie-Line Andreola, Fréderic Becquart, Wahbi Jomaa, Paul O. Verhoeven, Gérard Baldacchino, Simon Hemour, and D-Mask consortium

Inactivation test I Sample no	Mask (experiment no 3)	Negative control (uninfected cells)	Positive control (infected cells)
1	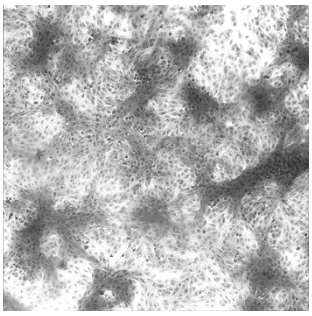	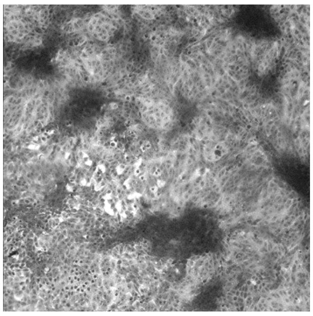	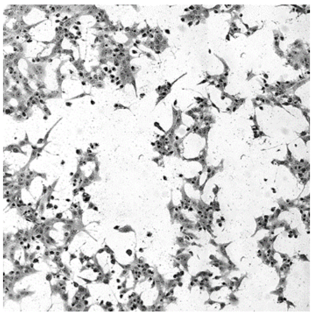
2	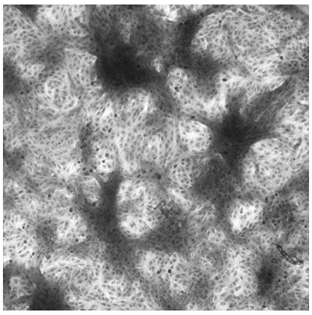	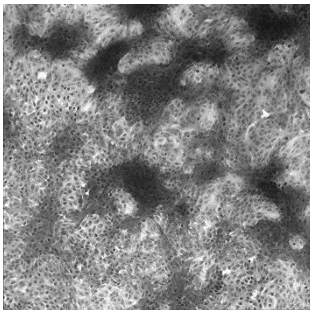	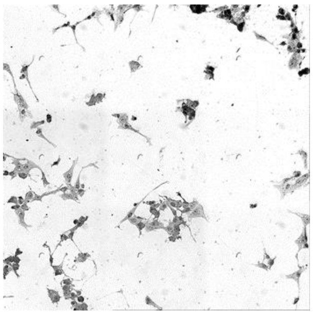
3	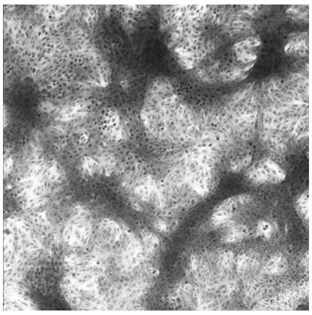	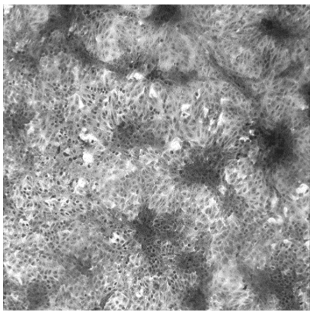	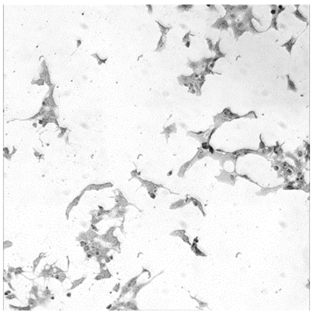
4	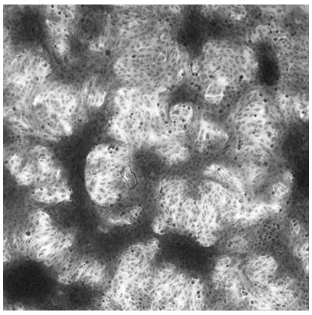	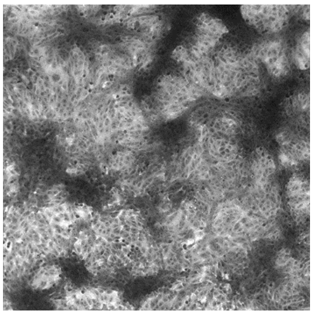	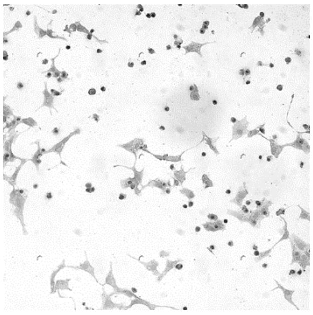
5	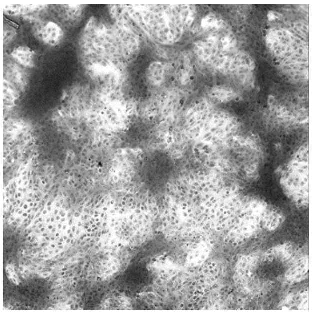	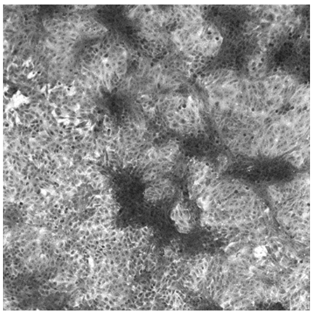	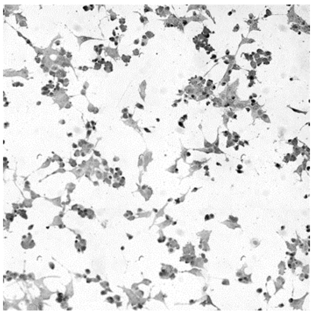

Figure S3. VeroE6 cells at day 6 post infection. (mask #3).
Vero E6 cells inoculated with SARS-CoV-2 recovered from paper discs after heat-inactivation (mask #3). Vero E6 growth with complete medium (negative control). Vero E6 cells inoculated with SARS-CoV-2 recovered from paper discs without heat-inactivation (positive control). Images were acquired at 40x magnification. Each image corresponds to 9 merged fields which cover approximately 0.95 mm².
