# Supplementary material for: The properties of hot household hygroscopic materials and their potential use for non-medical facemask decontamination
Source: PLoS One. 2021 Sep 7;16(9):e0255148. doi: 10.1371/journal.pone.0255148 (PMC8423240; doi:10.1371/journal.pone.0255148)
Supplement: S4 Fig — VeroE6 cells inoculated with SARS-CoV-2 recovered from paper discs after heat-inactivation (mask #4). VeroE6 growth with complete medium (VERO cells). Uninfected VeroE6 cells with paper discs after heat-inactivation (Negative control). VeroE6 cells inoculated with SARS-CoV-2 recovered from paper discs without heat-inactivation (positive control). (PDF) [file pone.0255148.s005.pdf]

Supporting Information S1
Marie-Line Andreola, Fréderic Becquart, Wahbi Jomaa, Paul O. Verhoeven, Gérard Baldacchino, Simon Hemour, and D-Mask consortium

Inactivation test II<br>Day	VERO cells	Negative control<br>(uninfected cells)	Positive control<br>(infected cells)
Day 0	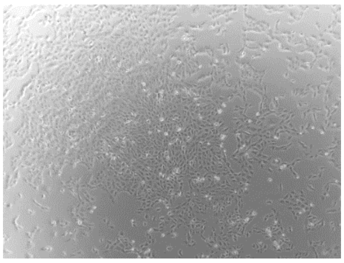		
Day 2		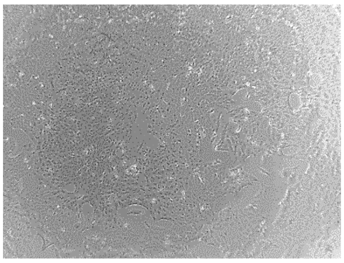	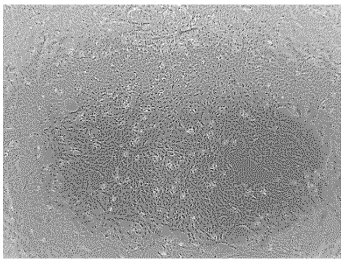
Day 7	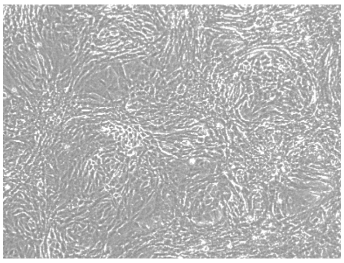	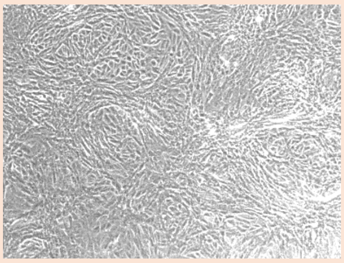	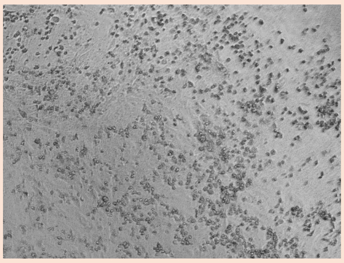

Inactivation test II<br>Day	Sample no 1<br>(experiment no 4)	Sample no 2<br>(experiment no 4)	Sample no 3<br>(experiment no 4)
Day 2	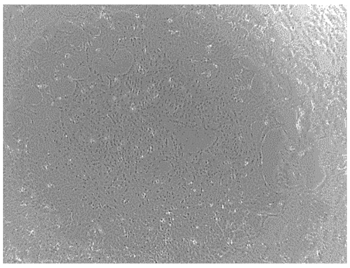	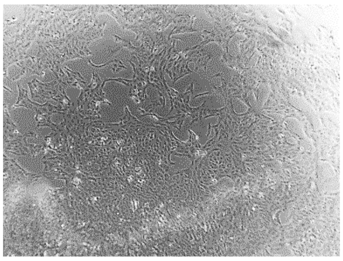	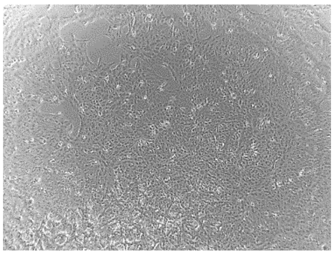
Day 7	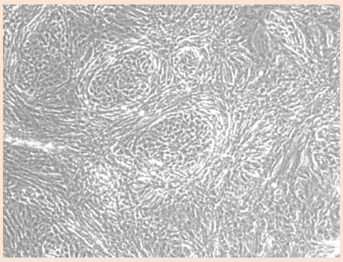	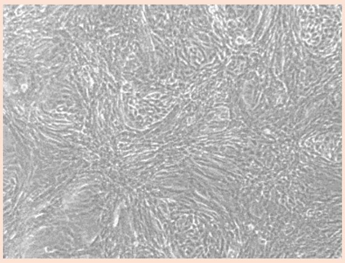	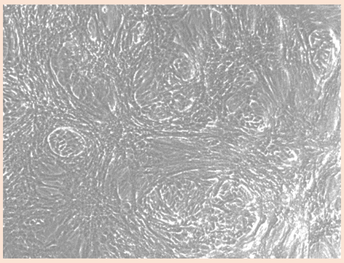

(image selected for the brief report are highlighted in orange)

Figure S4 VeroE6 cells at day 2 and 7 post infection. (mask #4)

VeroE6 cells inoculated with SARS-CoV-2 recovered from paper discs after heat-inactivation (mask #4). VeroE6 growth with complete medium (VERO cells). Uninfected VeroE6 cells with paper discs after heat-inactivation (Negative control). VeroE6 cells inoculated with SARS-CoV-2 recovered from paper discs without heat-inactivation (positive control).
