# Supplementary material for: The properties of hot household hygroscopic materials and their potential use for non-medical facemask decontamination
Source: PLoS One. 2021 Sep 7;16(9):e0255148. doi: 10.1371/journal.pone.0255148 (PMC8423240; doi:10.1371/journal.pone.0255148)
Supplement: S5 Fig — Methods from the experiment shown in S4 Fig. After centrifugation, 200 μl of supernatant were extracted using the « High Pure Viral Nucleic Acid » kit from Roche following the supplier’s recommendations. qRT-PCR was performed following indications of the supplier (SARS-CoV-2 RT-qPCR Perkin Elmer). Briefly, 6 μl of reactional mix were mixed with 14 μl of a 1/10 dilution of each sample. Amplification of positive and negative controls supplied in the kit were also performed. Two viral genes, ORF1ab-gene and N-gene, were targeted. According to the supplier, Ct ≤32 for ORF1ab or ≤35 for N-gene are considered as positive. Ct ≥40 or undetermined are negative. S5 Fig presents a representative experiment of qRT-PCR. Supernatant of cells infected with positive control (untreated virus) were positive with Ct of 15.80 and 16.10 for ORF1ab-gene and N-gene respectively. All other samples, uninfected cells, cells infected with heat treated samples, negative control of the kit, were undetected. Positive controls of the kit is detected with Ct ≤32 as expected: 29.25 for of ORF1ab and 28.92 for N-gene. (PDF) [file pone.0255148.s006.pdf]

Supporting Information S1
Marie-Line Andreola, Fréderic Becquart, Wahbi Jomaa, Paul O. Verhoeven, Gérard Baldacchino, Simon Hemour, and D-Mask consortium

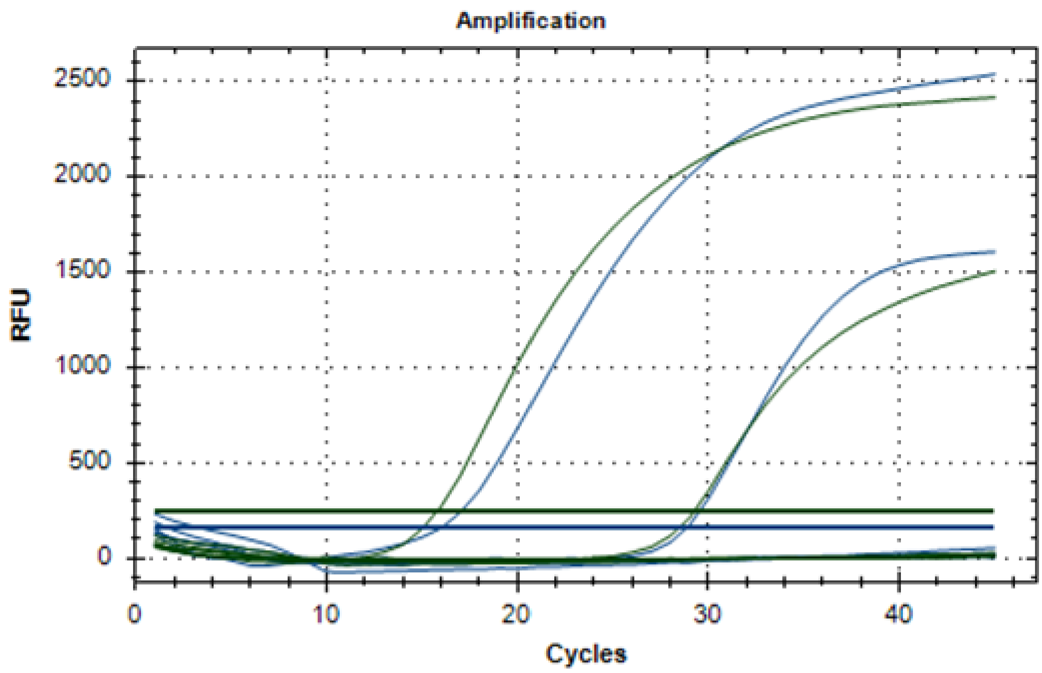

Figure S5 qRT-PCR amplification of viral RNA from the experiment shown in Fig. S4. Ct<20, untreated samples. Ct >20, treated samples.

Methods from the experiment shown in S4 Fig. After centrifugation, 200 µl of supernatant were extracted using the « High Pure Viral Nucleic Acid » kit from Roche following the supplier's recommendations. qRT-PCR was performed following indications of the supplier (SARS-CoV-2 RT-qPCR Perkin Elmer). Briefly, 6 µl of reactional mix were mixed with 14 µl of a 1/10 dilution of each sample. Amplification of positive and negative controls supplied in the kit were also performed. Two viral genes, ORF1ab-gene and N-gene, were targeted. According to the supplier, Ct ≤32 for ORF1ab or ≤35 for N-gene are considered as positive. Ct ≥40 or undetermined are negative. Figure S5 presents a representative experiment of qRT-PCR. Supernatant of cells infected with positive control (untreated virus) were positive with Ct of 15.80 and 16.10 for ORF1ab-gene and N-gene respectively. All other samples, uninfected cells, cells infected with heat treated samples, negative control of the kit, were undetected. Positive controls of the kit is detected with Ct ≤32 as expected: 29.25 for of ORF1ab and 28.92 for N-gene.
